# Screening and surveillance of hepatocellular carcinoma by serum des‐gamma‐carboxy prothrombin in patients with glycogen storage disease type Ia

**DOI:** 10.1002/jmd2.12414

**Published:** 2024-05-29

**Authors:** A. B. Schreuder, R. J. Overduin, N. C. Peltenburg, L. de Boer, F. A. J. A. Bodewes, T. G. J. Derks

**Affiliations:** ^1^ Department of Metabolic Diseases, Beatrix Children's Hospital University Medical Center Groningen, University of Groningen Groningen The Netherlands; ^2^ Department of Metabolic Diseases and internal medicine Erasmus Medical Center Rotterdam The Netherlands; ^3^ Department of Metabolic Diseases, Amalia Children's Hospital Radboud University Medical Center Nijmegen The Netherlands; ^4^ Department of Pediatric Hepatology and Gastroenterology, Beatrix Children's Hospital University Medical Center Groningen, University of Groningen Groningen The Netherlands

**Keywords:** des‐gamma‐carboxy prothrombin, glycogen storage disease type I, hepatocellular adenoma, hepatocellular carcinoma, tumor markers

## Abstract

No sensitive tumor marker for hepatocellular carcinoma (HCC) is available for patients with glycogen storage disease type Ia (GSDIa), in whom alpha‐fetoprotein and carcino‐embryonic antigen levels often remain normal. We describe increased levels of the HCC tumor marker des‐gamma‐carboxy prothrombin (DCP) in GSDIa patients with HCC. In one case DCP levels normalized after liver transplantation. We recommend including DCP as a screening HCC tumor marker in the surveillance of patients with GSDIa.

**Synopsis**

Des‐gamma‐carboxy prothrombin (DCP) is a screening tumor marker for HCC in the surveillance of GSDIa patients.

## 1 INTRODUCTION

Glycogen storage disease type Ia (GSDIa; OMIM #232200) is a rare inherited disorder of carbohydrate metabolism caused by mutations in the *G6PC1* gene, resulting in glucose‐6‐phosphatase (G6Pase) deficiency.^1^ Classical presenting symptoms during early childhood include severe fasting intolerance, failure to thrive, and hepatomegaly, but adult GSDIa patients exhibiting attenuated hypoglycemic phenotypes are increasingly recognized, as recently discussed.^2^ Strict medically prescribed dietary treatment aims to maintain euglycemia, and to prevent secondary metabolic perturbations and chronic complications, while preserving quality of life as much as possible. Despite intensive dietary treatment, serious long‐term complications occur, such as hepatocellular adenoma (HCA) and hepatocellular carcinoma (HCC).

The European Study on Glycogen Storage Disease Type I guidelines originate from 2002 and recommend screening for malignant transformation from HCA to HCC by imaging (ultrasound and MRI‐scan) and by serum alpha‐fetoprotein (AFP) and carcino‐embryonic antigen (CEA).^3^ The more recent GSDI practice guideline of the American College of Medical Genetics and Genomics acknowledges that there is no effective tumor marker, since AFP and CEA levels are often normal despite HCC.^4^

Des‐gamma‐carboxy prothrombin (DCP, also known as elevated protein induced vitamin K absence or antagonist‐II [PIVKA‐II]) is recognized as the most useful tumor marker in predicting HCC.^5^ It differentiates HCC from non‐malignant liver diseases. Moreover, it has been demonstrated that a combined analysis of DCP and AFP can improve screening for HCC in early stages.^6^

To the best of our knowledge, to date, only two case reports reported elevated DCP levels in GSDIa patients with confirmed HCC.^7^, ^8^ We hereby present two additional cases of GSDIa patients who developed HCC, in whom we observed increased DCP levels, despite normal levels of AFP and CEA. In one of these patients, DCP levels completely normalized following liver transplantation.

## 2 PATIENTS

**FIGURE 1:**
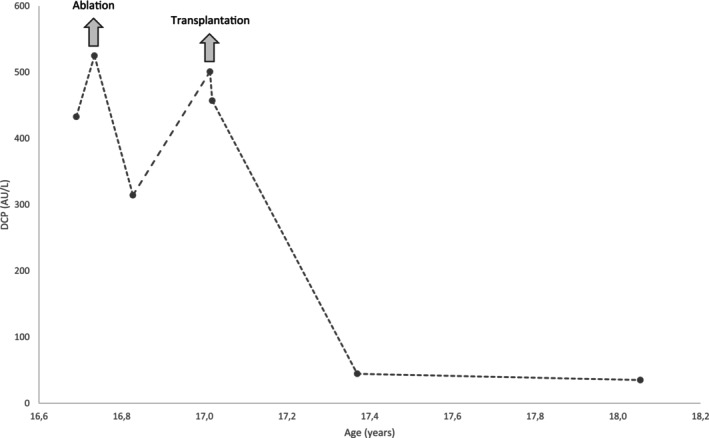
Course of serum des‐gamma‐carboxy prothrombin of patient‐1.

Table 1 presents the general characteristics and tumor markers in two GSDIa patients who developed HCC. Patient 1 displayed a decrease of DCP levels after ablation and normalization after liver transplantation (Figure 1).

**TABLE 1:** General characteristics and tumor markers in two GSDIa patients who developed HCC.

	Patient‐1	Patient‐2
Gender	M	F
Age at GSDIa diagnosis	Neonatally	4 years
Ethnicity	Turkish	Chinese
*G6PC1* variants		
*Allele 1*	c.809G > T (p.Gly270Val)	c.248G > A (p.Arg83His)
*Allele 2*	c.809G > T (p.Gly270Val)	c.648G > T (p.Tyr202Ter)
Age at HCC diagnosis	16 years (mode: histology)	20 years (mode: imaging)
Tumor markers at HCC diagnosis		
AFP, normal < 10 μg/L	3.8 μg/L	1.5 μg/L
CEA, normal 0.5–5.0 μg/L	0.8 μg/L	0.8 μg/L
DCP, normal 7.4–50.9 AU/L	457.1 AU/L	147.0 AU/L
Follow‐up	Liver transplantation	Listed for liver transplantation

Abbreviations: AFP, alpha‐fetoprotein; CEA, carcino‐embryonic antigen; DCP, des‐gamma‐carboxy‐prothtrombin.

## 3 DISCUSSION

The development of HCA and HCC poses a significant risk in patients with GSDIa, even with standard‐of‐care dietary management. The challenge lies in the screening and surveillance of HCC in GSDIa patients, as the conventional tumor markers for HCA and HCC exhibit low sensitivity in this patient group. In this context, we present two GSDIa patients with HCC, in whom elevated DCP levels were observed. Importantly, these elevated DCP levels returned to normal following tumor reduction interventions, such as ablation and liver transplantation.

To date, there are no GSDIa patient cohort studies or registries that systematically studied *G6PC1* genotype–phenotype relations, environmental risk‐factors, or ethnicities in GSDIa patients who developed HCC. While all GSDIa patients are considered at risk for developing HCC, homozygosity for the common Japanese c.648G > T (p.Tyr202Ter) *G6PC1* pathogenic splice variant has been associated with an even further increased risk.^1^ This variant is the predominant variant detected in GSDIa patients from Japanese and Korean descent. In our case series, patient 2 is compound heterozygote for this specific variant, and the c.248G > A (p.Arg83His) variant, which is the second most common *G6PC1* variant among patients in China and Taiwan.^9^

After Liebman et al. first described DCP as a serum marker of HCC, the marker has initially been widely used in Japan for HCC diagnosis and surveillance.^10^, ^11^ Two case reports that originate from Japan mentioned DCP as a tumor marker specifically for HCC screening in GSDIa patients. Sakamoto et al. reported a 21‐year‐old male GSDIa patient with HCC, in whom slightly elevated DCP levels (107 mAU/ml) were observed, whereas AFP and CEA both were normal. In their 19‐year‐old female GSDIa patient with HCC in whom AFP and CEA levels were normal, Okata et al. observed a DCP level of 208 mAU/ml.^8^ Both case reports did not mention the *G6PC1* variants in their patients. Our two cases show the same lack of elevation of AFP and CEA, but increases of DCP, which may suggest increased sensitivity of DCP to detect HCC. To the best of our knowledge, tumor markers are not included in the experimental studies of GSDIa animal models which develop HCC.^14^ Future preclinical and clinical research may focus on combinations of traditional and innovative HCC tumor markers (such as circulating exosomal microRNAs^12^) to improve early diagnosis and surveillance of HCC in GSDIa patients.

The hepatosteatosis in GSDIa, due to accumulation of glycogen and triglycerides, might underlie liver tumor formation, given that previous research has shown increased risk of HCC linked to steatosis in patients with non‐cirrhotic non‐alcoholic fatty liver disease.^13^ Macroautophagy is impaired in G6Pase‐deficient hepatic cells, both in vitro and in vivo.^14^ The deficiency in autophagy has been linked to hepatosteatosis in GSDIa, and the reversal of hepatosteatosis has been linked to the induction of autophagy in mice with GSDIa.^14^ Additionally, metabolic perturbations in GSD Ia associated with the loss of several cellular defenses, such as autophagy, antioxidant enzymes, dysregulation of endoplasmatic reticulum stress responses, and apoptosis, can lead to the formation of HCC.^15^

Next to the existing risk of GSDIa patients for developing HCC, increased incidence of HCC has been reported in murine studies after AAV gene delivery, of which some demonstrated that insertional mutagenesis by recombinant AAV vectors occurred.^16^ Interestingly, Cho et al. showed that when liver‐specific G6pc‐knockout mice that develop HCA/HCC are treated at the tumor‐developing stage with rAAV‐G6PC, this prevents de novo HCA/HCC development.^17^ After completing the phase 1–2 study of adeno‐associated viral (AAV) serotype 8 gene delivery in adult GSDIa patients (NCT03517085) and a phase 3 study (NCT05139316), patients are subsequently entering long‐term follow‐up studies (such as NCT03970278). Therefore, in addition to the existing guidelines, our observations are not only valuable for healthcare providers following GSDIa patients, but also for designing long‐term follow‐up studies after innovative therapies.

In conclusion, our findings suggest that DCP levels can be elevated in GSDIa patients with HCC. We advocate for the inclusion of DCP as a screening tumor marker for HCC in the surveillance of GSDIa patients. However, to strengthen our observations, further data collection from larger patient cohorts is essential.

## AUTHOR CONTRIBUTIONS

Andrea Schreuder wrote the paper with support of Terry Derks. Terry Derks conceived the original idea. All authors provided critical feedback and helped shape the research, analysis, and manuscript.

## FUNDING INFORMATION

The authors received no financial support for the research, authorship, and/or publication of this article.

## CONFLICT OF INTEREST STATEMENT

Andrea Schreuder declares that she experiences no competing interest concerning the content of this manuscript. However, in the past 36 months, there have been confidentiality agreements with third parties. For all private–public relationships, all contracts are via UMCG Contract Research Desk, and all payments are to UMCG. Ruben Overduin declares that he experiences no competing interests concerning the content of this manuscript. There is a consultation agreement with Ultragenyx Pharmaceutical Inc of which the contract is via UMCG Contract Research Desk and all payments are to UMCG. Chantal Peltenburg declares that she experiences no competing interest concerning the content of this manuscript. However, she is an independent investigator for study sponsored by Clinuvel. Lonneke de Boer declares that she experiences no competing interest concerning the content of this manuscript. Frank Bodewes declares that he experiences no competing interests concerning the content of this manuscript. Terry Derks declares that he experiences no competing interests concerning the content of this manuscript. However, there are confidentiality agreements with third parties. In the past 36 months, there have been consultation agreements (with Danone, Ultragenyx Pharmaceutical Inc, ModernaTX Inc, and Beam Therapeutics), contracts for financial research support for investigator‐initiated research (NCT04311307) and sponsor‐initiated research (NCT03517085, NCT03970278, NCT05139316, and NCT05196165), honoraria for lectures or presentations (by MEDTalks, Prelum, and Danone), and participations in a Data Safety Monitoring Board (NCT05095727) and Advisory Boards (Ultragenyx Pharmaceutical Inc, ModernaTX Inc, and Beam Therapeutics). For all private‐public relationships, all contracts are via UMCG Contract Research Desk, and all payments are to UMCG.

## ETHICS STATEMENT

Ethics approval was not required; consent from the patients was obtained for publication.

## PATIENT CONSENT STATEMENT

Additional informed consent was obtained from all patients for which identifying information is included in this article.

## Data Availability

Data supporting the results reported in this article are available via the corresponding author.
